# Assessment of the Mineral Composition and the Selected Physicochemical Parameters of Dietary Supplements Containing Green Tea Extracts

**DOI:** 10.3390/foods11223580

**Published:** 2022-11-10

**Authors:** Justyna Ośko, Adrian Szewczyk, Paulina Berk, Magdalena Prokopowicz, Małgorzata Grembecka

**Affiliations:** 1 Department of Bromatology, Faculty of Pharmacy, Medical University of Gdańsk, Gen. J. Hallera Avenue 107, 80-416 Gdańsk, Poland; 2 Department of Physical Chemistry, Faculty of Pharmacy, Medical University of Gdańsk, Gen. J. Hallera Avenue 107, 80-416 Gdańsk, Poland; 3 Student Scientific Circle at the Department of Bromatology, Faculty of Pharmacy, Medical University of Gdańsk, Gen. J. Hallera Avenue 107, 80-416 Gdańsk, Poland

**Keywords:** dietary supplements, elements, PTMI, quality, factor analysis, physicochemical parameters

## Abstract

Our aim was to assess the mineral composition as well as the physicochemical quality of green tea-based dietary supplements (capsules and tablets) with respect to average weight, size and shape, friability, breaking force and disintegration time. Products were analysed by flame atomic absorption spectrometry for Ca, K, Mg, Na, Cr, Cu, Fe, Zn, Mn, Pb and Cd. Approximately 60% of the analysed supplements met the requirements of European and Polish regulations. The dietary supplements passed weight variation tests, but not all products had compliant capsule sizes. One product in tablet form failed the friability test, and eleven dietary supplements in tablet form failed the disintegration test. The supplements were characterised by a varied elemental composition, with the highest values found for Ca, Fe, Zn and Mn. The recommended daily allowance realisation for Fe and Zn in two dietary supplements (capsule form) exceeded 100%. As a result of the estimation of the monthly intake of toxic metals, it was concluded that the tested products do not pose a risk to consumer health. Significant relationships (*p* < 0.001) were found for K, Mg, Na, Cu, Fe, Mn and Zn. The application of factor and cluster analyses allowed the verification of the similarity of green tea extracts contained in dietary supplements to the natural raw material, green tea leaves, used as a reference material.

## 1. Introduction

Today, dietary supplements constitute an integral part of the global food market. Because they are categorised as food products, and subjected to food law, they are popular products with general availability. According to statistics conducted by Statista Consumer Market Outlook in 2019, almost 60% of respondents in Poland take dietary supplements on a daily basis [1]. Polish consumers spent more than U.S. $1.4 billion on supplements in 2020 [2]. In turn, Poland was in 8th place (U.S. $449 million) among the 25 countries assessed in 2021 in terms of revenue from the sale of dietary supplements containing vitamins and minerals [3]. Due to the great availability of dietary supplements, there has been a continuous increase in the consumption of these products, which has also been driven by the COVID-19 pandemic [4]. Many patients, when looking for new means to reduce the risk of SARS-CoV-2 infection, usually chose supplements containing vitamins C and D and zinc (Zn), hoping these would protect against viral infection or reduce the risk of an aggressive course of the disease [5,6,7]. In China and the USA, it was found that customers were notably interested in supplements, and their sales increased significantly [7,8,9]. Despite the availability of vaccines, the demand for dietary supplements during a pandemic continues to increase [4]. 

The requirements for dietary supplements in Poland are not strictly specified, resulting in easier product launches. Consequently, newer products are appearing in a fairly short time. However, despite their often-questionable origin, they are willingly chosen by consumers. Their choices are motivated by the desire to improve their health, well-being, appearance, or even to reduce body weight. Consumers are encouraged to purchase foodstuffs through persuasive advertising, the perception of which may result in confusion between the effect of supplement intake and the effect of a drug. At the same time, lack of information on their contraindications and side effects may indicate to the patient that these preparations are safer than medicines [10]. The main reasons for confusing a supplement with a medicine are the visual similarity of the packaging and the dosage form of both preparations. In addition, supplements readily available in Poland do not require a prescription, and can be purchased not only in a pharmacy but also in a supermarket or on the Internet. Consumers may be more inclined to purchase these preparations if they are more readily available [11]. 

Polish legal standards, in contrast to those in force in the United States of America (USA), do not impose significant requirements on dietary supplements, except that they must not have a pharmacological effect and should be in the form of dosage preparations: tablets, capsules, dragees, sachets with powder, bottles with droppers or ampoules with liquid [12,13]. In the USA, the technology for manufacturing dietary supplements is comparable to that for pharmaceutical products. As dietary supplements and pharmaceutical products come in similar dosage forms, there are corresponding quality requirements. For example, the last print edition of the *United States Pharmacopoeia* (USP 43-NF 38) [14] contains information on acceptable weight variation [15], friability [16], breaking force of tablets [17] and disintegration time [18] of dietary supplements. In the European Union (EU), dietary supplements, unlike medicinal products, are not subjected to pharmacovigilance [19] or to requirements for the determination of physicochemical parameters, which often has serious consequences for consumers. Manufacturers are also not obliged to demonstrate quality and batch-to-batch homogeneity in the same way as for medicinal products. This makes it difficult to detect adulteration [19]. The use of preparations of inadequate quality can result in numerous side effects and the occurrence of many problems related to their intake [13]. Incorrect intake of medicines or dietary supplements can result in an excess of drug or supplement components in the body. Some patients have difficulty swallowing medication due to a history of illness, e.g., stroke, Huntington’s disease, oesophageal cancer, Parkinson’s disease or multiple sclerosis [20]. Very often, such people develop dysphagia characterised by problems swallowing food and even saliva, which can affect the intake of oral medication [21]. For this reason, the correct form of the supplement, its shape and size and texture are as important as the vitamins and minerals contents [20]. 

Supplements containing plant extracts have become particularly popular, as they are considered by patients safer and healthier than synthetic drugs [22]. Manufacturers declare that their preparations contain a certain amount of a plant extract, but this information says nothing about the actual content of bioactive substances or minerals. Discrepancies between the declared and real content of the extract used, or essential elements in one dose, can have a significant impact on human health and might lead to an ineffective supplementation or an overdosage. The composition and mineral content of natural plants can vary depending on climate, temperature, season, soil and other factors [23]. A number of techniques are currently in use to assess a product’s authenticity. The most commonly used techniques for the analysis of chemical composition of dietary supplements based on plant extracts is liquid chromatography coupled to a detector (UV/Vis) or photodiode array (PDA) [24,25], or a charged aerosol detector (CAD, Corona detector) [26], gas chromatography with a flame ionization detector (FID) [27] or mass spectrometry with a triple quadrupole coupled with gas chromatography (GC-MS/MS) [28]. Studies dealing with the analysis of metals have used inductively coupled plasma mass spectrometry (ICP-MS) [29,30] or atomic absorption spectrometry (AAS) [31,32]. The determination of trace elements in plants by ICP-MS or FAAS allows rapid analysis with good precision and accuracy. Results from published works show that dietary supplements based on plant extracts contained elements in the μg/g range, and their concentrations varied widely [33]. The use of appropriate statistical methods has frequently aided in the interpretation of the results. In many studies, they have become an excellent tool to extend information about the samples [34,35,36].

In the presented study, the selected green tea-based dietary supplements were assessed in terms of (1) their mineral composition and toxic metals contamination, (2) the products’ physicochemical parameters in view of the US Pharmacopoeia (USP 43-NF 38) requirements, (3) and correct labelling according to Polish and European legislation. The application of appropriate chemometric methods, such as factor and cluster analyses, allowed the verification of the similarity of green tea extracts contained in dietary supplements to the natural raw material, green tea (*Camellia sinensis*) leaves, used as a reference material.

## 2. Materials and Methods

The study material consisted of 35 selected dietary supplements containing green tea extracts (twenty-one different manufactures), purchased from online pharmacies in Poland. The characteristics of products are presented in Table A1. The analysed materials consisted of 26 supplements in capsule form and nine in tablet form. The products were selected for their current availability, content of the main ingredient, green tea extract, and the form for which physicochemical parameters could be determined according to the USP and Food and Drug Administration (FDA) [37]. The products purchased were from the EU countries and the USA. Five of the dietary supplements analysed did not have country of origin declared on the label.

### 2.1. Average Weight

The dietary supplements tested were subjected to a weight variability test, which is described in USP Guideline 43-NF 38; <2091> chapter *Weight variability of dietary supplements* [15]. The weight of 20 individual tablets and capsules was measured on an analytical balance with a resolution of ±0.0001 g (Semi-micro balance TS2215Di, VWR, Leuven, Belgium). The average weight for each formulation was calculated. Based on the USP [15], the highest and lowest mass values of each capsule were between 90% and 110% of the mean capsule mass. For tablets, the following limits were set: the mass values of no more than two out of twenty individual tablets weighed could vary by more than ±5% of the mean mass, and no tablet could vary by more than ±10% of the mean tablet mass.

### 2.2. Shape and Size

Due to the lack of guidelines for analysing the size and shape of the fixed dosage form of dietary supplements, an assessment of the shape and size of dietary supplements was made based on the work published by Overgaard et al. [37]. To assess shape and size, 20 individual capsules and tablets were measured for each test preparation using a digital calliper (measuring range: 0–150 mm, resolution: 0.01 mm, Parkside, OWIM GmbH & Co. KG, Neckarsulm, Germany) model: HG00962A (0.01 mm). For capsules, length and width (=depth) were measured, length (=width) and depth for round tablets, and width, length and depth for oval or oblong tablets. Tablets with length = width were classified as round. Industry guidelines published by the FDA for the size and shape of capsules and tablets were also used to evaluate the supplements analysed [38].

### 2.3. Friability Test

The friability of tablets was conducted following the USP 43-NF 38 guidelines; <1216> chapter *Tablet friability* [16]. For tablets with a unit weight greater than 650 mg, 10 whole tablets were tested. For tablets with a unit weight equal to or lower than 650 mg, a sample of whole tablets corresponding to approx. 6.5 g was used. Dust-free tablets were placed in a pharmacopeia friabilator (Erweka TAR 10, Erweka, Warsaw, Poland) and rotated for 4 min at 25 rpm. The friability (*F*) was calculated using Equation (1):

(1) $$F=\frac{w_{1}-w_{2}}{w_{1}}\times 100\%$$

where *w*_1_ and *w*_2_ are the masses (g) of the tablets before and after the test, respectively. Tablets, for which friability was ≤1%, met the pharmacopeia requirements. Broken, cracked, or cleaved tablets failed the test, despite the F value.

### 2.4. Breaking Force Test

The breaking force that causes tablets to fracture was determined using a hardness tester (Erweka TBH 125, Erwerka, Warsaw, Poland) following the USP 43-NF 38 guideline; <1217> chapter “Tablet breaking force” [17]. The measurement was carried out for 10 samples of each dietary supplement in the form of tablets and expressed as an average value ± standard deviation. 

### 2.5. Disintegration Time

The disintegration time of investigated dietary supplements was performed in agreement with the USP 43-NF 38 <2040> chapter *Disintegration and dissolution of dietary supplements* [18] using a pharmacopeia disintegration apparatus (Erweka ZT 320, Erweka, Warsaw, Poland) which raises and lowers the basket in the immersion fluid at a constant frequency rate of 29–32 cycles per min. As an immersion fluid, 0.1 M HCl (pH = 1.0, T = 37 ± 2 °C) was used to simulate the conditions of the stomach. Six individual tablets or capsules of each dietary supplement were investigated. The following pharmacopeia limit was set: after 30 min of the test, all of the investigated tablets and capsules should be completely disintegrated. In the case of capsules, non-disintegrated fragments of the capsule could be observed only if their content was completely wetted after 30 min of the test. 

### 2.6. Preparation of Samples for Flame Atomic Absorption Spectroscopy (FAAS) Analysis

In quartz crucibles, 5 g of each test dietary supplement was weighed on an analytical balance ±0.0001 g (Semi-micro balance TS2215Di, VWR, Leuven, Belgium) in triplicate.

The tested dietary supplements were mineralised using a dry technique in an electric oven (Lindberg/Blue M, model no. BF51828C, Asheville, NC, USA) with a temperature gradient of up to 540 °C. The resulting material was handled according to the procedure described by Brzezicha-Cirocka et al. [39] Blank samples were measured for each measuring series, following the same procedure as for the analysed samples.

### 2.7. Elemental Analysis

A Thermo Scientific iCE 3000 series atomic absorption spectrometer (Thermo Fisher Scientific, Waltham, MA, USA) with flame atomisation (FAAS) was applied for the determination of the elements Na, K, Ca, Mg, Mn, Fe, Zn, Cr, Cu, Cd, Pb. Sodium and K were determined using a 0.2% caesium chloride (Merck, Darmstadt, Germany) solution as an ionisation buffer, while for the determination of Ca and Mg, a 0.4% lanthanum oxide (Merck, Darmstadt, Germany) solution was used as a correction buffer [40].

The limit of detection (*LOD*) and quantification (*LOQ*) of the method were determined according to the formula proposed by Konieczka and Namieśnik [41]:

(2) $$LOD=\bar{x}+3SD$$

(3) LOQ=3LOD

where $\bar{x}$ is average of the blank and *SD* is standard deviation.

In order to assess the accuracy and precision of the analytical procedure, the certified reference material, Oriental Basma Tobacco Leaves INCT-OBTL-5 (Institute of Nuclear Chemistry and Technology, Laboratory of Nuclear Analytical Method, Warsaw, Poland), was investigated. The recovery value of the analysed elements ranged from 89 to 112%, and the precision from 0.02% to 10.2%. The validation parameters of the method are presented in Table A2.

### 2.8. Statistical Analysis

The data obtained were presented as average values ± standard deviations (SD). The Shapiro-Wilk test was used to assess the normality of the data distribution at a significance level of *p* < 0.05. The occurrence of statistically significant differences between the manufacturer’s declaration weight data and the obtained capsule/tablet weight was performed using Student’s *t*-test. The Kruskal-Wallis, non-parametric test, Spearman rank correlation, factor (FA), and cluster analyses (CA) were used to analyze element analysis data in dietary supplements and green tea leaves, which data was served as reference material [39]. All statistical analyses were carried out using Statistica 13.3 (TIBCO Software Inc., Palo Alto, CA, USA).

### 2.9. Evaluation of the Tested Food Supplements According to Polish Legislation

Based on the Act of 25 August 2006 on food and nutrition safety [12], the dietary supplements tested were assessed in terms of producers’ declarations contained on the packaging. The assessment was performed taking into account the following requirements of Polish and European law:

1. According to Art. 48 of the Act [12], foodstuffs marketed on the territory of the Republic of Poland must be labelled in the Polish language.
2. In accordance with Regulation (EU) No 1169/2011 of the European Parliament and of the Council of 25 October 2011 on the provision of food information to consumers [42], products were assessed for the presence of the name of the food (Art. 17), the list of ingredients (Art. 18), substances or products causing allergies or intolerances (Art. 21); the quantification of ingredients (Art. 22), net quantity (Art. 23), the presence of a minimum durability date, a ‘use by’ date (Art. 24), storage conditions (Art. 25); country or places of origin (Art. 26), instructions for use (Art. 27), and nutritional information (Art. 29).
3. In accordance with the Regulation of the Minister of Health of 9 October 2007 on the composition and labelling of dietary supplements [43], the following requirements were assessed: occurrence of the term “dietary supplement”; name of the category of nutrients or substances characterising the product or an indication of their properties; portion of the product recommended for consumption during the day; warning not to exceed the recommended portion for consumption during the day; occurrence of a statement that dietary supplements may not be used as a substitute (replacement) for a varied diet; statement that dietary supplements should be kept out of the reach of small children (Art. 5 p. 2); the content of vitamins and minerals and other substances with a nutritional or other physiological effect in numerical form per recommended daily portion of the product, as well as information on the content of vitamins and minerals as a percentage of the nutrient reference values for daily intake (or a statement that no NRVs has been established).

## 3. Results and Discussion

**Figure 1:**
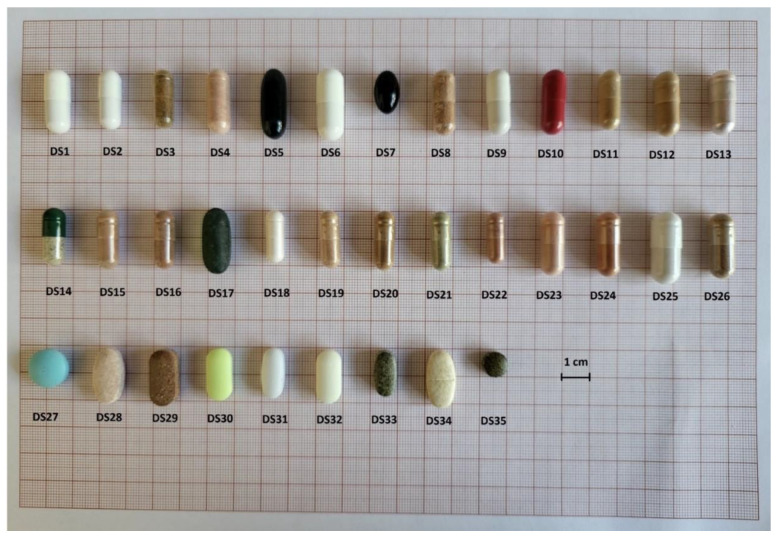
Variation of sizes and shapes of the analysed dietary supplements.

**Figure 2:**
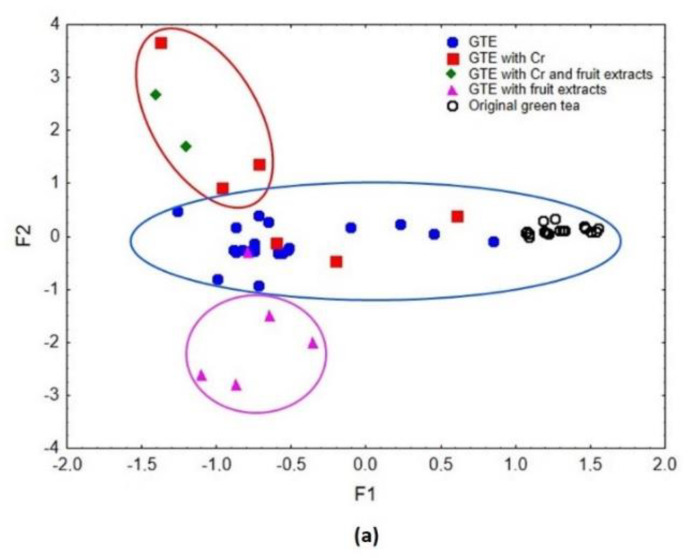

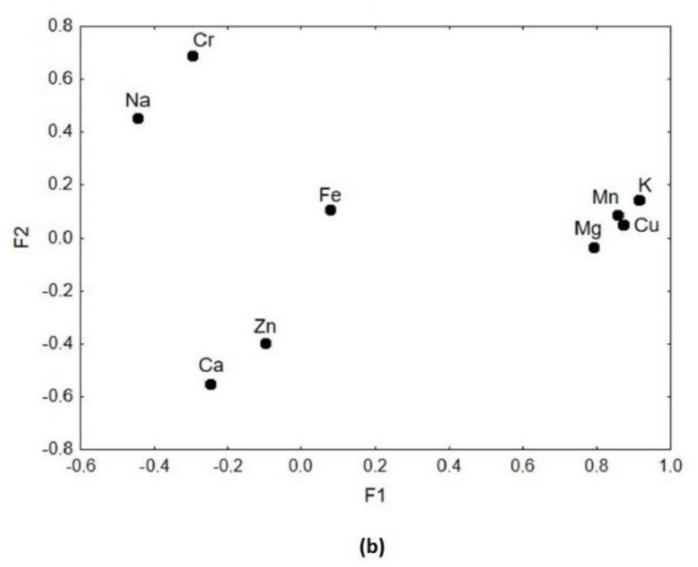
(**a**) Scatterplot of object samples for two factors of the analysed dietary supplements (GTE—dietary supplements containing green tea extracts. GTE with Cr—dietary supplements containing green tea extracts with chromium; GTE with Cr and fruit extracts—dietary supplements containing green tea extracts with chromium and fruit extracts; GTE with fruit extracts—dietary supplements containing green tea extracts with fruit extracts) and green tea samples. (**b**) Scatterplot of objects samples of two factors of the analysed dietary supplements and green tea samples. The data concerning green tea are based on Brzezicha-Cirocka et al. [39].

**Figure 3:**
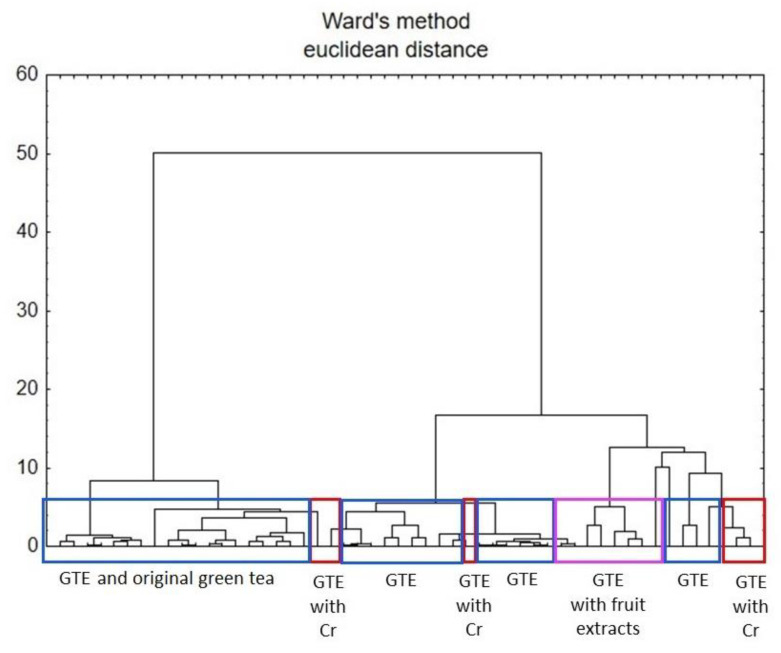
Hierarchical dendrogram for the analysed dietary supplements. GTE—dietary supplements containing green tea extracts; GTE with Cr—dietary supplements containing green tea extracts with chromium; GTE with Cr and fruit extracts—dietary supplements containing green tea extracts with chromium and fruit extracts; GTE with fruit extracts—dietary supplements containing green tea extracts with fruit extracts) and green tea samples. The data concerning green tea are based on Brzezicha-Cirocka et al. [39].

The results of the evaluation of physicochemical parameters such as size, shape, weight uniformity, disintegration time, friability and breaking force of the dietary supplements, are presented in Table 1 and Table 2 (for the capsule form) and Table 3 (for the tablet form). The tables also show the percentage of the manufacturer’s declaration in relation to the measured product weight. Figure 1 illustrates the size of the measured dietary supplements, the exact data for which can be found in Table 1. The elemental data of the analysed products are presented in Table 4. The results of the chemometric analysis carried out based on the mineral composition of dietary supplements and green tea are presented in Table 5 and Figure 2a,b and Figure 3. The recommended intake for individual elements according to Polish nutritional standards (Table 6) as well as the risk of human exposure to toxic elements in the tested products were also estimated (Table 7). The compliance of the manufacturer’s declaration for Cr content with the labelled value was assessed, as shown in Table 8.

### 3.1. Qualitative Evaluation of Dietary Supplements Containing Green Tea Extracts According to the United States Pharmacopoeia (USP) Guidelines

#### 3.1.1. Assessment of Weight Uniformity

The average weight of the capsules and tablets analysed was compared with the weight declared by the manufacturer and expressed as a percentage. The Min and Max values indicated the percentage relationship between the lowest measured value and the highest value in relation to the average capsule/tablet weight value. The results for capsules and tablets are shown in Table 2 and Table 3, respectively.

Based on Student’s *t*-test, there were no statistically significant differences between the average capsule/tablet weight (df = 30; t = −0.298; *p* = 0.768) and the manufacturer’s declaration for the 16 dietary supplements of 35 investigated (DS1-3, DS5-7, DS9-10, DS12-14, DS26-29 and DS31).

In accordance with the requirements of the pharmacopoeia weight variation study, two dietary supplements in capsule form (DS3 and DS14) had weight values of 20 weighed capsules close to the range of 90–110% of the average capsule weight, while the other products were within the specified range, meeting the specified requirements [15]. The dietary supplement DS5 had the highest mean weight of the analysed capsules, i.e., 1505 ± 7 mg, which was equivalent to 111% of the manufacturer’s declared weight (Table 2). In contrast, the smallest value of the average weight of the analysed capsules was 370 ± 18 mg in the DS3 product, representing 137% of the weight declared by the manufacturer. 

All tablet products passed the pharmacopoeia weight variation test (Table 3). The product with the highest mean weight of the tablets and of all the dietary supplements containing green tea extract analysed was the DS29 supplement (1497 ± 17). The percentage of weight declared by the manufacturer for this product was 105%. The lowest average mass value of all tablet products analysed was obtained for DS35 (336 ± 13 mg). However, this supplement did not have a declared tablet mass, making it impossible to estimate the degree of compliance with the measured value.

Uniformity in the weight of dosage form units ensures the product’s homogeneity. This is very important in order to introduce the same amount of active substances into the body when taking the same number of dosage form units [45]. All the supplements analysed were characterised by uniformity of weight, which indicated the reproducibility of their production. However, the average tablet weight of four products exceeded 1 g (Table 3), which might indicate problems with the intake of these preparations. Tablets with an average weight of more than 1 g may pose a problem with swallowing, especially for geriatric patients [20,46].

#### 3.1.2. Shape and Size

All dietary supplements in the form of capsules and tablets were compared in terms of size and shape (Table 1). Among the 35 preparations analysed, 26 were characterised by a cylindrical capsule shape. Among the dietary supplements in tablet form, two were round in shape. i.e., DS27 and DS35. Four products were in the form of oblong tablets, i.e., DS29, DS30, DS32 and DS33. The remaining three formulations were oval tablets, i.e., DS28, DS31 and DS34. The formulation with the largest size was DS25 (Figure 1).

The size and shape of capsules and tablets affect their ease of passage through the pharynx and oesophagus, which in turn can affect a patient’s ability to swallow preparations [47]. The US Food and Drug Administration [47] recommends that “the largest tablet or capsule size should not exceed 22 mm, and capsules should not exceed standard size 00”. The capacity of 00 capsules is 0.95 mL, and they are 23.3 ± 0.3 mm in length and 8.56 mm in diameter [47].

A study in adults assessing the relationship of the ease of swallowing tablets and capsules to their size showed an increase in swallowing difficulty in patients taking tablets larger than 8 mm in diameter [48,49,50]. The size of a tablet or capsule influenced oesophageal passage. Channer and Virjee [48] compared the passage time of 8 mm diameter round tablets with 11 mm diameter round tablets and 14 mm × 9 mm oval tablets. They found that tablets with smaller dimensions had a shorter passage time. Difficulty in swallowing tablets with a diameter of more than 8 mm is associated with a number of side effects such as pain and choking, which may result in patient non-compliance with taking the formulations in question [13].

All tablet formulations analysed met FDA recommendations. However, given the studies [48,49,50] suggesting that tablets with a diameter of more than 8 mm often exhibit swallowing problems, it can be concluded that all tablet formulations studied (DS27–DS35) may pose a problem when taken by patients. The capsule products DS5, DS6 and DS25 did not comply with the FDA’s capsule length and diameter requirements. In addition, dietary supplements DS7 and DS17 did not comply with the capsule diameter requirement. For capsule dimensions, 21 preparations were found to be comfortable to ingest.

#### 3.1.3. Breaking Force and Friability

Hardness and friability tests were performed for tablets (Table 3) to evaluate the mechanical strength and susceptibility to breakage, which are crucial factors during further operations, handling, transportation, and storage [51]. In the case of breaking force, the measured values were in the range of 80–474 N (Table 3); thus, they differed significantly between manufacturers. There are several factors that influence the final hardness of the obtained tablets, such as the characteristics of the powders/granules to be compressed, the type and amount of excipients used (e.g., lubricants, glidants, anti-adherents), and compression force [52]. It is well-known that the desired values of breaking force should be repeatable to provide good quality of the final product. Unfortunately, our studies demonstrated the scatter of breaking force values. In the case of five out of nine dietary supplements in the form of tablets investigated, the difference between the lowest and the highest reported values of breaking force was higher than 50 N (Table 3). Moreover, for the DS27, DS31, and DS32 tablets it exceeded 100 N. Thus, the manufacturing process and quality of the obtained tablets remain questionable. Considering the friability values (Table 3), only one of the dietary supplements examined did not meet the USP pharmacopeia requirement (DS28—friability value higher than 1.0%).

#### 3.1.4. Disintegration Time

The results of the disintegration time of capsules and tablets are presented in Table 3 and Table 4, respectively. Dietary supplements in the form of capsules disintegrated faster compared to tablets. Based on the manufacturers’ declaration, almost 70% of investigated capsules were composed of gelatine, whereas hydroxypropyl methylcellulose (hypromellose) was the main excipient in the remaining 30% of investigated products. Only three dietary supplements in the form of capsules did not pass the pharmacopeia disintegration test—after 30 min of the test the residual parts of the capsule shell were observed with dry content inside. The capsule shells of these three supplements were composed of hypromellose. It was shown that acidic conditions may impede the disintegration of hypromellose-based capsules, whereas the disintegration of gelatine-based capsules was not influenced by the pH of the medium [53,54]. During the production of dietary supplements, manufacturers use empty capsules purchased from other providers. It might be assumed that such capsules are standardized in most cases; thus, the obtained positive results of the disintegration test for capsules are both not surprising and in agreement with our previous results obtained for beetroot-based dietary supplements [13].

In the case of tablets, only one of the nine dietary supplements investigated met the pharmacopeia disintegration requirements (Table 3). Moreover, eight of the analysed supplements in the tablet form did not disintegrate even after continuation of the experiment for the additional 15 min (45 min in total). Suitable mechanical properties of tablets should allow for adequate disintegration during digestion [55]. It should be noted that both the proper design of tablets and their manufacture are more complex processes compared to the production of capsules. Thus, improper formulation of the tablet results in insufficient disintegration of dosage form and reduced dissolution of active substances. For example, in the case of the examined dietary supplements, such factors as lack of disintegrant or its insufficient concentration in the tablets, together with high breaking force values, can explain the obtained negative results of the disintegration test. The practical aspect of these results should also be considered. Tablets that do not disintegrate cannot be easily dispersed in a glass of water, which is the recommended procedure for patients who have problems with swallowing [56]. Based on the obtained results (Table 3), it can be concluded that most of the investigated tablets were designed inappropriately and are characterized by a lack of suitable quality.

### 3.2. Evaluation of the Selected Elements’ Contents in Green Tea—Based Dietary Supplements

Unfortunately, literature data on green tea-based dietary supplements are deficient; therefore, we compared the dietary supplements tested to a natural product, such as green tea leaves [41], in terms of mineral content.

#### 3.2.1. Results of Elemental Analysis

The analysed dietary supplements were characterised by a wide variation in the content of Ca, Cu, Mg, K, Na, Fe, Zn, Mn, Cd and Pb (Table 4). Among the macroelements, the highest average concentrations were determined for K (16.48 ± 0.13 mg/g) in the DS3 preparation, Na (8.95 ± 0.65 mg/g) in DS32, and Mg (2.88 ± 0.24 mg/g) and Ca (69.80 ± 4.76 mg/g) in the DS27 product. Malik et al. [57] obtained higher levels of K (20.3 mg/g) in dried green teas leaves, and lower (9.50 mg/g K) than McKenzie et al. [34]. However, in the case of Na, its average content in the dietary supplements (0.05–8.95 mg/g) was significantly higher than that determined in green tea by McKenzie et al. [34] (0.017–0.305 mg/g Na). The higher Na concentration in dietary supplements may be due to the addition of excipients such as sodium salts of carboxymethyl starch or carboxymethyl cellulose, which are found in the formulation of the DS32 supplement, among others. These substances act as a binders, coatings or disintegrants [58].

Dietary supplements containing green tea extracts were found to be a good source of Fe and Mn. The highest determined contents of Fe (4658 ± 20 µg/g) in DS5 and Mn (807 ± 17 µg/g) in DS21 were similar to the results obtained by Dambiec et al. [59] for black tea leaf samples. The Mn content in DS21 was comparable to the value determined in green tea by McKenzie et al. [34] (385–2081 µg/g). In the case of Zn, the highest concentration of this element was determined in the DS34 product (4465 µg/g). In contrast, the other dietary supplements analysed were characterised by a lower Zn content (<LOD-31.5 µg/g) and similar to the results determined in green tea obtained by Deka et al. (20.2–38.0 µg/g) [60]. The highest Cu content (30.71 ± 0.64 µg/g) was determined in DS14 and was higher than the results obtained by Deka et al. (12.6–22.7 µg/g) [60]. Due to the presence of declaration concerning Cr content in the eight products analysed (Table 4), these supplements constituted a good source of this element except for DS28. The highest Cr concentration was found in DS8 (76.71 µg/g).

The content of the toxic metals, Pb and Cd, was determined in a total of 19 dietary supplements (Pb in eight products and Cd in eleven products). The remaining dietary supplements contained Pb and Cd below the LOD = 0.08 and 0.01 µg/g, respectively (Table 4). Lead content was in the range <LOD-1.01 µg/g, with the highest values obtained for the DS21 supplement. A similar Pb content in green tea leaves was determined by Ma et al. [61]—0.87 µg/g. Lower values were obtained by Hamza et al. [62] (0.04–0.13 µg/g Pb). Similarly, a lower Pb content in supplements containing the plant component was determined by Augustsson et al. [63] at 0.16 µg/g. In the case of Cd, its content was in the range <LOD-0.31 µg/g, with the highest value found in in the DS35 supplement. The determined Cd content in the analysed products was higher than that determined in the green tea leaves, i.e., 0.007–0.02 µg/g [62] and 0.04 µg/g [61]. The supplements containing the ground up plant component (berries, herbs) contained 0.05 µg Cd/g [61].

#### 3.2.2. Results of Chemometric Analysis

The non-parametric Spearman’s rank test was used at three significance levels, i.e., *p* < 0.05, *p* < 0.01 and *p* < 0.001 for the dietary supplements and green tea samples, which data were taken from our previously published paper [39]. Elemental analysis data of original green teas published by Brzezicha-Cirocka et al. [39] constituted a reference material for the analysed dietary supplements containing green tea extracts. They were used for the Kruskal-Wallis test (*p* < 0.005), Dunn’s test, FA and CA analysis. Negative and positive correlations were found between the analysed elements. The positive correlations (*p* < 0.001) were found in the database of all the analysed samples between the following pairs of elements: K-Mg, K-Cu, K-Fe, K-Mn, K-Zn, Mg-Cu, Mg-Mn, Mg-Zn, Ca-Zn, Cu-Fe, Mn-Cu, Cu-Zn, Fe-Mn, Fe-Zn and Mn-Zn. Negative relationships (*p* < 0.001) were determined for K-Na, Mg-Na, Cu-Na, Mn-Na and Zn-Na.

A statistical analysis using the Kruskal-Wallis test showed statistically significant differences in the analysed database, categorised by composition of the products: green tea extracts (GTE), green tea extracts with Cr (GTE with Cr), green tea extracts with fruits extracts (GTE with fruit extracts), green tea extracts with Cr and fruit extracts (GTE with Cr and fruit extracts) and original green tea leaves [39].

The relationships between the categories of products and their elemental compositions were as follows: K (H = 32.979; *p* = 0.000), Mg (H = 24.648; *p* = 0.000), Na (H = 35.085; *p* = 0.000), Ca (H = 9.600; *p* = 0.048), Cr (H = 19.522; *p* = 0.001), Cu (H = 29.897; *p* = 0.000), Fe (H = 25.779; *p* = 0.000), Mn (H = 34.352; *p* = 0.000) and Zn (H = 29.385; *p* = 0.000).

Next, the post hoc Dunn’s test was conducted at three levels of significance (*p* < 0.05; *p* < 0.01 and *p* < 0.001) to determine significant differences between particular categories. The results are presented in Table 5. Significant relationships (*p* < 0.001) were found for K, Mg, Na, Cu, Fe, Mn or Zn and GTE, GTE with Cr and original green tea [39].

Factor analysis allowed the dietary supplement samples to be differentiated in terms of similarity to natural green tea, Cr enrichment, and fruit extract additives used. The results are shown in Figure 2a,b. The elements included in the analysis were K, Mg, Na, Ca, Mn, Fe, Cu, Zn and Cr. The explained variance by the first factor (F1) amounted to 36.8%, and the second factor (F2) to 13.2%. Both factors cumulatively explained 50.1% of the total variance, whereas the eigenvalues for F1 and F2 were 3.31 and 1.19, respectively.

As can be observed in Figure 2a, F1 was responsible for the differentiation of samples based on their similarity to the natural product—green tea [39]. The level of resemblance to the reference samples increased with the F1 value. We can assume that the closer the supplement samples were distributed to the natural product (green tea) [39], the more the main ingredient (green tea extracts) was authentic or present in greater quantity. The highest F1 values corresponded to green tea samples [39] distinguished by K, Mg, Mn and Cu (Figure 2b). Factor 1 also discriminated dietary supplements containing green tea extracts in a dominant proportion from supplement samples with fruit extracts or enriched in Cr. Lower F1 values corresponded to dietary supplement samples containing added fruit, which were characterised by Ca and Zn, and dietary supplement samples enriched in Cr, described by Cr and Na. Factor 2 made it possible to distinguish between dietary supplements’ samples with a dominant proportion of fruit extracts (low F2 values), samples containing green tea extracts (GTE) and natural green tea (medium F2 values), and samples enriched in Cr (high F2 values) (Figure 2a). The lowest F2 values characterising dietary supplement samples with added fruit extracts related to Ca and Zn content. Supplement samples containing GTE and green tea corresponded to Fe, K, Mg, Mn and Cu content. In contrast, the highest F2 values responsible for the separation of samples enriched in Cr, were related to Cr and Na (Figure 2b).

Cluster analysis (CA) was carried out based on Ward’s method using the Euclidean distance measure (Figure 3). The dendrogram shows the distribution of green tea samples [39] and green tea-based dietary supplements (including fruit extracts and Cr enrichment). In Figure 3, a dendrogram consisting of eight main clusters corresponding to different groups of samples is shown. This analysis was similar to the factorial analysis separation.

Both chemometric techniques allowed the assessment of the dietary supplements’ quality in view of their composition, especially the proportion of the main ingredient compared to the reference material, which was natural green tea [39].

#### 3.2.3. Assessment of the Intake of Selected Elements

The daily recommended intake suggested by the manufacturer of each supplement was compared to the current Polish standards [44] and presented as their percentage of actual intake (RDA). Due to the lack of a specific RDA for K, Na and Mn, the actual adequate intake (AI) was calculated for these elements [44]. Table 6 shows the percentage of realisation of the RDA and AI for each element of all dietary supplements tested (capsules/tablets), in the form of a range (min-max), assuming the product is taken at the recommended daily dose (as recommended by the manufacturer, Table A1).

Among dietary supplements in both capsule and tablet form, the highest percentage of intake realisation (above 100%) was recorded for Mn, Zn and Fe (Table 6). Within the capsule formulations, the highest percentage of realisation of dietary recommendations was determined for Fe for men (<LOD-280%), and for Zn for women (<LOD-235%). The lowest percentage of realisation of sufficient intake was obtained for the elements: Na and K (Table 6). For the tablet form, the highest percentage of AI realisation was found for Mn, both for women, which was 29.50%, and for men, 23.10%. Among the macroelements, the highest percentage of RDA realisation was determined for Ca (15.10% for both women and men).

#### 3.2.4. Assessment of Exposure of Toxic Elements

The exposure to toxic metals, such as Pb and Cd, as a result of the consumption of the selected green tea-based dietary supplements was also assessed. Lead content was below the LOD (LOD = 0.08 µg/g) in 16 products out of 35, while Cd content was below the LOD (LOD = 0.01 µg/g) in 24 formulations. An estimation of Cd intake was made in 11 dietary supplements and compared with the provisional tolerable monthly intake (PTMI) of 25 µg/kg body weight, established by the Scientific Committee on Food [64].

The estimation was made for a person weighing 70 kg, based on the consumption of recommended daily intake (Table A1) per 30 days (Table 7). In the case of the PTWI for Pb, which was previously established by the Scientific Committee on Food, and amounted to 25 µg/kg b.w., no such evaluation was made, as this dose was withdrawn in the 73rd Expert Report of the FAO/WHO Committee on Food Additives [65]. In view of numerous scientific reports, it was concluded that a new weekly intake limit for Pb could not have been established and, thus, the previous PTWI was no longer updated [65,66].

It was concluded that there is no risk of exposure to this element from daily consumption of these products at the recommended dose as the PTMI. However, the dietary supplement DS35 was characterised with the highest percentage of PTMI for Cd (Table 7). Consumption of this product for 30 days at the manufacturer’s recommended dosage resulted in a Cd exposure of 9.45% PTMI. Therefore, consumption of other Cd-containing food products along with this supplement may result in an increased risk of exposure to this element.

#### 3.2.5. Evaluation of the Manufacturer’s Declaration of Analysed Minerals

The dynamically developing market for dietary supplements requires special oversight. This is evidenced by a study conducted by the Polish controlling body, i.e., the Supreme Chamber of Control. The findings of the audit showed that a proper level of safety of dietary supplements is not ensured in Poland [67]. An under or over supply of minerals present in foods can cause adverse health effects [68]. The mineral content declared by the manufacturer may differ between the actual and the declared content by +45/−20% [64]. To assess the compliance of the dietary supplements analysed, the percentage of the manufacturer’s declaration was calculated for each of the analysed products that had such a declaration (Table 8). Among the dietary supplements analysed, only eight had a manufacturer’s declaration of Cr content. The percentage of the manufacturer’s declaration ranged from 16.1% to 164%. Two products had Cr content above the declared value. The preparation with the highest declared Cr content (DS8) had a much higher level of Cr amounting to 151% of the manufacturer’s declaration. Despite the manufacturer’s declaration of Cr content (5 µg/capsule) in DS28, its percentage was not verified, due to Cr content below the limit of detection (LOD < 0.17 µg/g). The percentage of the manufacturer’s declaration of all Cr-containing products was not within the acceptable difference (+ 45/−20%).

Based on the Student’s *t*-test, there were no statistically significant differences between the Cr average content (df = 14; t = −0.608; *p* = 0.552) and the manufacturer’s declaration for the eight dietary supplements (DS1, DS8, DS9, DS10, DS28, DS30, DS32 and DS34).

As a general rule, for the purpose of determining whether a substance constitutes a significant amount, the Regulation [43] specifies that 15% of the nutrient reference intake values (NRVs) for a given ingredient per portion, if the package contains only one portion, should be met. The nutrient reference value for chromium amounts to 40 µg. This condition was met for five supplements out of eight products containing such a declaration. Dietary supplements DS9, DS28 and DS34 did not meet the requirements of the regulation regarding the Cr content of 15% NRVs.

### 3.3. Evaluation of Compliance of the Information on the Supplement’s Packaging Based on Current Polish Legislation

The dietary supplements tested were reviewed in accordance with Article 48 of the Food and Nutrition Safety Act in terms of the requirement to label foodstuffs in the Polish language [12]. As a result of the verification, it was found that only one product, DS34, out of all the supplements tested did not have labelling in Polish [12]. It was found that 2.9% of the supplements did not have a statement that the dietary supplement cannot be used as a substitute for a varied diet, 5.7% of the analysed products did not contain information on special storage conditions, and 8.6% did not have warning on storage out of the reach of children. Moreover, 11.4% of the dietary supplements lacked information about the country of origin on the packaging. Of the products, 34.3% did not contain information on the vitamin and mineral content as a percentage of the reference daily intake values (or no indication on the label that the NRVs was not set). All guidelines for the correct labelling of supplements can be found in the Regulation of the European Parliament and of the Council (EU) No 1169/2011 [42] and the Regulation of the Minister of Health on the composition and labelling of dietary supplements [43].

## 4. Conclusions

Approximately 60% of the analysed dietary supplements containing green tea extracts met the requirements of Regulation (EU) No. 1169/2011 of the European Parliament and the Council, as well as the Polish regulations. Due to the lack of European regulations concerning the weight and size of the pharmaceutical form of the dietary supplement, an assessment was made following the USP 43-NF 38 and FDA. All products, both in capsules and tablets, met the requirements of the pharmacopoeia weight variation test. In contrast, three capsule products did not fulfil the form size requirements. Considering fragility test values, only one of the dietary supplements tested did not meet the USP pharmacopoeia requirement. Moreover, eleven of the tested products had a disintegration time of more than 30 min, and failed the pharmacopoeia test. Not only could some of the analysed supplements cause difficulties in ingestion due to their improper size, but in the case of tablets, their disintegration time was also unacceptable in terms of quality requirements.

The dietary supplements tested were also characterised by a varied elemental composition. Based on evaluation of the intake of the dietary supplements studied, the percentage of realisation of the RDA for Fe and Zn (capsules) was found to exceed 100%. Human exposure to toxic metals, such as Cd, as a result of consumption of the product was also estimated. It was concluded that there was no risk associated with Cd exposure by consuming the tested supplements according to the manufacturer’s recommended dosage for a period of 30 days. However, additional consumption of other products may increase this risk significantly. Based on the evaluation of the manufacturer’s declaration in terms of compliance of the weight of the pharmaceutical form, two products were found to have a significantly higher unit weight, as with Cr content, which was found to be higher than labelled. In addition, three supplements did not meet the requirements of the regulation regarding content of this element at 15% NRVs.

Chemometric techniques allowed the assessment of the quality, and therefore authenticity, of the dietary supplements in terms of the green tea extracts they contained. Based on factor and cluster analyses, it was possible to evaluate the supplements in view of the similarity of the green tea extract contained in them to the natural raw material, and to make a distinction between products enriched in Cr and those dominated by fruit extracts.

## Figures and Tables

**Table 1:** Analysis of shape and size of the analysed dietary supplements.

Code	Shape	Length ± SD [mm]	Width ± SD [mm]	Depth ± SD [mm]	W + L + D [mm] ^1^	FDA Recommendation ^2^
DS1	cylindrical capsule	23.10 ± 0.04	8.30 ± 0.05	8.30 ± 0.05	40	acceptable
DS2	cylindrical capsule	21.51 ± 0.06	7.42 ± 0.04	7.42 ± 0.04	36	acceptable
DS3	cylindrical capsule	21.21 ± 0.07	7.46 ± 0.04	7.46 ± 0.04	36	acceptable
DS4	cylindrical capsule	23.34 ± 0.08	8.34 ± 0.05	8.34 ± 0.05	34	acceptable
DS5	cylindrical capsule	25.42 ± 0.15	9.01 ± 0.05	9.01 ± 0.05	43	unacceptable
DS6	cylindrical capsule	25.51 ± 0.09	9.72 ± 0.06	9.72 ± 0.06	45	unacceptable
DS7	cylindrical capsule	15.11 ± 0.23	9.15 ± 0.11	9.15 ± 0.11	33	unacceptable
DS8	cylindrical capsule	23.33 ± 0.08	8.40 ± 0.04	8.40 ± 0.04	40	acceptable
DS9	cylindrical capsule	23.12 ± 0.05	8.34 ± 0.07	8.34 ± 0.07	40	acceptable
DS10	cylindrical capsule	22.61 ± 0.16	8.33 ± 0.07	8.33 ± 0.07	39	acceptable
DS11	cylindrical capsule	21.40 ± 0.09	7.44 ± 0.04	7.44 ± 0.04	36	acceptable
DS12	cylindrical capsule	23.23 ± 0.08	8.34 ± 0.05	8.34 ± 0.05	40	acceptable
DS13	cylindrical capsule	23.04 ± 0.15	8.27 ± 0.06	8.27 ± 0.06	39	acceptable
DS14	cylindrical capsule	21.35 ± 0.10	7.48 ± 0.04	7.48 ± 0.04	36	acceptable
DS15	cylindrical capsule	21.91 ± 0.42	7.53 ± 0.04	7.53 ± 0.04	37	acceptable
DS16	cylindrical capsule	21.32 ± 0.10	7.48 ± 0.02	7.48 ± 0.02	36	acceptable
DS17	cylindrical capsule	23.60 ± 0.25	9.54 ± 0.17	9.54 ± 0.17	43	unacceptable
DS18	cylindrical capsule	19.10 ± 0.15	6.79 ± 0.04	6.79 ± 0.04	33	acceptable
DS19	cylindrical capsule	21.01 ± 0.20	7.44 ± 0.08	7.44 ± 0.08	36	acceptable
DS20	cylindrical capsule	21.72 ± 0.06	7.60 ± 0.04	7.60 ± 0.04	37	acceptable
DS21	cylindrical capsule	23.33 ± 0.17	8.31 ± 0.04	8.31 ± 0.04	40	acceptable
DS22	cylindrical capsule	18.71 ± 0.14	6.67 ± 0.07	6.67 ± 0.07	32	acceptable
DS23	cylindrical capsule	23.32 ± 0.34	8.39 ± 0.02	8.39 ± 0.02	40	acceptable
DS24	cylindrical capsule	23.31 ± 0.09	8.37 ± 0.03	8.37 ± 0.03	40	acceptable
DS25	cylindrical capsule	25.84 ± 0.12	9.78 ± 0.07	9.78 ± 0.07	45	unacceptable
DS26	cylindrical capsule	21.81 ± 0.22	7.49 ± 0.06	7.49 ± 0.06	37	acceptable
DS27	round tablet	13.82 ± 0.04	13.81 ± 0.04	7.57 ± 0.16	35	acceptable
DS28	oval tablet	19.70 ± 0.03	10.20 ± 0.02	10.2 ± 0.02	40	acceptable
DS29	oblong tablet	20.22 ± 0.05	9.14 ± 0.01	7.78 ± 0.03	37	acceptable
DS30	oblong tablet	18.71 ± 0.02	8.72 ± 0.02	5.52 ± 0.03	33	acceptable
DS31	oval tablet	18.12 ± 0.04	8.10 ± 0.02	5.61 ± 0.08	32	acceptable
DS32	oblong tablet	18.80 ± 0.03	8.78 ± 0.03	6.40 ± 0.03	34	acceptable
DS33	oblong tablet	16.21 ± 0.03	8.13 ± 0.02	6.17 ± 0.08	30	acceptable
DS34	oval tablet	20.42 ± 0.03	10.31 ± 0.02	6.45 ± 0.02	37	acceptable
DS35	round tablet	9.13 ± 0.02	9.13 ± 0.02	4.66 ± 0.18	23	acceptable

^1^ Sum of length, width and depth; ^2^ FDA recommends that “the largest tablet or capsule size should not exceed 22 mm, and capsules should not exceed 23.3 ± 0.3 mm in length and 8.56 mm in diameter”.

**Table 2:** Physical parameters of the dietary supplements in capsule form.

Code	Weight Uniformity						Disintegration Time	
	Declared Weight [mg]	Average Weight ± SD [mg]	Percentage of Declaration [%]	Min [%] ^1^	Max [%] ^2^	Pharmacopeia Criteria	Disintegration Time [min]	Pharmacopeia Criteria
DS1	524	545 ± 6	104	99	102	passed	23:20	passed
DS2	380	388 ± 10	102	94	104	passed	29:30	passed
DS3	270	370 ± 18	137	90	108	passed	9:30	passed
DS4	NA	809 ± 65	ND	95	103	passed	25:00	passed
DS5	1350	1505 ± 7	111	99	101	passed	15:40	passed
DS6	1220	1251 ± 15	102	96	102	passed	21:20	passed
DS7	700	714 ± 78	102	97	102	passed	25:10	passed
DS8	NA	786 ± 14	ND	96	103	passed	30:00^3^	passed
DS9	700	637 ± 26	91	91	104	passed	12:50	passed
DS10	722	743 ± 8	103	96	105	passed	12:20	passed
DS11	NA	632 ± 10	ND	97	103	passed	10:20	passed
DS12	589	624 ± 26	106	92	108	passed	5:10	passed
DS13	650	661 ± 35	102	99	101	passed	9:30	passed
DS14	433	393 ± 21	91	90	112	passed	14:30	passed
DS15	NA	604 ± 6	ND	94	103	passed	28:00	passed
DS16	NA	624 ± 13	ND	96	106	passed	30:00 ^3^	passed
DS17	NA	1390 ± 19	ND	98	105	passed	30:00 ^3^	passed
DS18	NA	493 ± 17	ND	94	106	passed	30:00 ^3^	passed
DS19	NA	619 ± 12	ND	94	103	passed	30:00 ^3^	passed
DS20	NA	608 ± 8	ND	98	103	passed	>30:00 ^4^	failed
DS21	NA	657 ± 18	ND	94	104	passed	7:15	passed
DS22	NA	474 ± 6	ND	98	102	passed	30:00 ^3^	passed
DS23	NA	896 ± 21	ND	96	106	passed	>30:00 ^4^	failed
DS24	NA	954 ± 7	ND	99	102	passed	>30:00 ^4^	failed
DS25	NA	984 ± 19	ND	98	105	passed	14:00	passed
DS26	617	760 ± 9	123	97	103	passed	8:30	passed

NA—not available, no information on package; ND—no data; value cannot be calculated due to lack of data from the manufacturer; ^1^ percentage specified for the lowest mass measurement values; ^2^ percentage specified for the highest mass measurement values; ^3^ non-disintegrated parts of capsule shell observed with completely wetted content inside; ^4^ non-disintegrated parts of capsule shell observed with not completely wetted content inside.

**Table 3:** Physical parameters of the dietary supplements in tablet form.

Code	Weight Uniformity						Disintegration Time		Friability		Breaking Force		
	Declared Weight [mg]	Average Weight ± SD [mg]	Percentage of Declaration [%]	Min [%] ^1^	Max [%] ^2^	Pharmacopeia Criteria	Disintegration Time [min]	Pharmacopeia Criteria	Value [%]	Pharmacopeia Criteria	Average Value ± SD [N]	Min (N)	Max (N)
DS27	980	1013 ± 13	103	98	103	passed	>30:00	failed	0.0	passed	198 ± 39	144	259
DS28	1100	1114 ± 4	101	99	101	passed	>30:00	failed	8.7	failed	86 ± 5	76	94
DS29	1422	1497 ± 17	105	98	103	passed	>30:00	failed	0.1	passed	385 ± 15	356	402
DS30	NA	785 ± 8	ND	99	103	passed	>30:00	failed	0.0	passed	474 ± 19	447	497
DS31	675	717 ± 5	106	98	102	passed	>30:00	failed	0.0	passed	291 ± 39	227	331
DS32	NA	950 ± 10	ND	99	103	passed	>30:00	failed	0.0	passed	396 ± 38	339	463
DS33	NA	846 ± 23	ND	93	104	passed	>30:00	failed	0.2	passed	200 ± 23	148	224
DS34	NA	1050 ± 8	ND	99	102	passed	10:30	passed	0.0	passed	103 ± 2	101	108
DS35	NA	336 ± 13	ND	92	108	passed	>30:00	failed	0.0	passed	80 ± 8	68	93

NA—not available, no information on package; ND—no data; value cannot be calculated due to lack of data from the manufacturer; ^1^ percentage specified for the lowest mass measurement values; ^2^ percentage specified for the highest mass measurement values.

**Table 4:** Content of the selected elements in the analysed dietary supplements [mg/g and µg/g].

	K [mg/g]	Mg [mg/g]	Na [mg/g]	Ca [mg/g]	Cr [µg/g]	Cu [µg/g]	Fe [µg/g]	Mn [µg/g]	Zn [µg/g]	Pb [µg/g]	Cd [µg/g]
DS1	1.11 ± 0.01	0.391 ± 0.009	0.450 ± 0.003	0.13 ± 0.04	25.61 ± 1.98	0.36 ± 0.03	5.43 ± 0.42	2.25 ± 0.04	0.76 ± 0.46	<LOD	<LOD
DS2	0.08 ± 0.01	0.74 ± 0.03	0.52 ± 0.001	0.16 ± 0.06	0.201 ± 0.009	0.12 ± 0.03	7.45 ± 0.71	0.53 ± 0.02	1.59 ± 0.23	<LOD	<LOD
DS3	16.48 ± 0.13	1.44 ± 0.15	0.99 ± 0.07	2.59 ± 0.29	2.83 ± 0.06	6.61 ± 0.03	445 ± 35	245 ± 5	31.5 ± 19.9	0.440 ± 0.001	<LOD
DS4	1.43 ± 0.13	1.43 ± 0.12	0.57 ± 0.05	0.06 ± 0.04	0.30 ± 0.04	0.110 ± 0.007	10.11 ± 0.64	28.82 ± 2.88	0.99 ± 0.15	<LOD	<LOD
DS5	0.201 ± 0.001	0.062 ± 0.002	0.27 ± 0.07	0.15 ± 0.03	1.82 ± 0.05	0.11 ± 0.03	4658 ± 20	7.43 ± 0.17	<LOD	<LOD	<LOD
DS6	0.25 ± 0.02	0.461 ± 0.002	0.37 ± 0.02	0.13 ± 0.15	<LOD	0.15 ± 0.01	14.72 ± 4.79	0.63 ± 0.02	0.73 ± 0.01	0.111 ± 0.003	<LOD
DS7	6.95 ± 0.20	0.182 ± 0.007	0.64 ± 0.03	<LOD	<LOD	1.83 ± 0.02	<LOD	35.90 ± 2.41	2.46 ± 0.09	<LOD	<LOD
DS8	3.071 ± 0.007	0.443 ± 0.002	1.82 ± 0.02	0.49 ± 0.08	76.71 ± 4.48	0.34 ± 0.02	11.01 ± 0.30	7.26 ± 0.37	1.31 ± 0.87	<LOD	0.03 ± 0.02
DS9	14.42 ± 0.88	4.05 ± 0.04	1.31 ± 0.11	0.34 ± 0.06	7.73 ± 0.42	7.28 ± 0.33	12.18 ± 0.23	8.50 ± 0.16	4.47 ± 0.16	<LOD	0.010 ± 0.004
DS10	2.00 ± 0.34	0.173 ± 0.001	3.03 ± 0.42	0.15 ± 0.01	26.42 ± 2.66	0.28 ± 0.04	496 ± 41	11.30 ± 1.92	0.48 ± 0.53	<LOD	<LOD
DS11	0.481 ± 0.001	0.442 ± 0.001	1.28 ± 0.14	30.13 ± 1.75	1.54 ± 0.02	0.38 ± 0.03	38.81 ± 0.06	9.08 ± 0.13	1.16 ± 0.05	<LOD	0.01 ± 0.02
DS12	0.631 ± 0.007	0.29 ± 0.01	0.541 ± 0.004	0.53 ± 0.03	0.55 ± 0.11	0.552 ± 0.005	14.78 ± 0.19	138.0 ± 4.27	3.59 ± 0.10	<LOD	<LOD
DS13	0.07 ± 0.03	1.32 ± 0.04	0.38 ± 0.03	0.24 ± 0.11	<LOD	0.19 ± 0.06	8.41 ± 1.38	3.83 ± 0.15	1.09 ± 0.25	<LOD	<LOD
DS14	4.18 ± 0.08	0.57 ± 0.06	0.79 ± 0.05	1.66 ± 0.07	0.53 ± 0.12	30.71 ± 0.64	120 ± 1	344 ± 11	10.01 ± 0.53	0.76 ± 0.02	0.09 ± 0.14
DS15	1.12 ± 0.04	0.15 ± 0.01	0.320 ± 0.001	0.48 ± 0.09	0.32 ± 0.02	0.77 ± 0.01	19.78 ± 0.25	88.22 ± 4.41	2.50 ± 0.28	0.08 ± 0.01	<LOD
DS16	0.96 ± 0.17	0.0510 ± 0.0001	0.36 ± 0.03	0.13 ± 0.05	0.35 ± 0.11	1.38 ± 0.04	9.29 ± 0.74	10.21 ± 1.59	2.01 ± 0.05	<LOD	<LOD
DS17	0.87 ± 0.02	0.030 ± 0.001	0.20 ± 0.01	0.02 ± 0.01	<LOD	8.98 ± 0.52	1.94 ± 0.30	0.13 ± 0.05	<LOD	<LOD	<LOD
DS18	1.09 ± 0.01	1.20 ± 0.02	0.34 ± 0.04	0.25 ± 0.04	<LOD	1.051 ± 0.004	<LOD	3.41 ± 0.03	4.47 ± 0.15	<LOD	<LOD
DS19	9.39 ± 0.68	0.30 ± 0.06	2.33 ± 0.72	2.43 ± 1.60	<LOD	0.17 ± 0.02	9.16 ± 2.32	120 ± 0.38	3.03 ± 0.25	0.09 ± 0.03	<LOD
DS20	1.76 ± 0.03	0.310 ± 0.001	0.81 ± 0.04	0.24 ± 0.01	0.70 ± 0.07	2.11 ± 0.04	16.51 ± 0.48	27.41 ± 0.46	2.09 ± 0.07	<LOD	<LOD
DS21	11.51 ± 1.14	2.42 ± 0.03	0.44 ± 0.05	4.74 ± 0.11	1.07 ± 0.15	13.01 ± 0.14	2.91 ± 0.13	807 ± 17	28.71 ± 9.38	1.01 ± 0.03	0.07 ± 0.01
DS22	0.45 ± 0.01	0.78 ± 0.05	2.28 ± 0.07	0.27 ± 0.01	0.42 ± 0.04	0.23 ± 0.05	8.99 ± 0.68	14.10 ± 0.09	2.67 ± 0.73	<LOD	0.02 ± 0.06
DS23	1.01 ± 0.02	0.010 ± 0.002	0.41 ± 0.04	<LOD	<LOD	<LOD	1.92 ± 0.57	1.97 ± 0.24	<LOD	<LOD	<LOD
DS24	0.191 ± 0.001	0.10 ± 0.05	0.28 ± 0.01	0.11 ± 0.05	0.32 ± 0.09	0.07 ± 0.02	6.25 ± 1.89	9.24 ± 0.07	1.93 ± 0.17	<LOD	<LOD
DS25	0.23 ± 0.04	1.02 ± 0.01	6.12 ± 0.83	24.31 ± 0.76	<LOD	0.11 ± 0.01	10.92 ± 0.57	7.50 ± 0.65	1.07 ± 0.17	<LOD	<LOD
DS26	15.61 ± 0.63	0.98 ± 0.02	1.30 ± 0.01	0.47 ± 0.02	<LOD	0.99 ± 0.02	41.4 ± 16.2	242 ± 3	5.40 ± 0.02	0.10 ± 0.01	<LOD
DS27	4.67 ± 0.01	2.88 ± 0.24	0.82 ± 0.11	69.80 ± 4.76	1.46 ± 0.18	1.64 ± 0.14	7.64 ± 0.26	35.01 ± 0.69	4.02 ± 0.27	0.15 ± 0.01	<LOD
DS28	0.01 ± 0.01	2.83 ± 0.02	0.05 ± 0.01	0.07 ± 0.07	<LOD	0.05 ± 0.02	3.520 ± 0.004	0.90 ± 0.06	<LOD	<LOD	<LOD
DS29	0.23 ± 0.02	0.84 ± 0.02	0.280 ± 0.002	23.61 ± 0.29	0.54 ± 0.01	0.33 ± 0.02	15.01 ± 1.01	130 ± 8	3.41 ± 0.34	<LOD	<LOD
DS30	6.69 ± 0.45	0.91 ± 0.06	0.22 ± 0.02	0.79 ± 0.04	35.7 ± 2.19	0.45 ± 0.06	13.42 ± 1.45	3.45 ± 0.28	4.53 ± 0.32	<LOD	0.02 ± 0.08
DS31	6.36 ± 1.31	0.95 ± 0.13	0.72 ± 0.09	2.90 ± 0.59	0.20 ± 0.03	0.37 ± 0.01	11.63 ± 0.74	3.92 ± 0.34	5.14 ± 0.21	<LOD	0.05 ± 0.14
DS32	5.52 ± 0.04	0.632 ± 0.005	8.95 ± 0.65	0.23 ± 0.05	16.66 ± 0.30	0.49 ± 0.02	7.31 ± 0.52	63.13 ± 0.21	1.96 ± 0.05	<LOD	<LOD
DS33	2.78 ± 0.44	0.42 ± 0.05	0.34 ± 0.01	89.01 ± 9.80	0.78 ± 0.15	0.46 ± 0.06	<LOD	3.32 ± 0.13	1.67 ± 0.31	<LOD	0.09 ± 0.01
DS34	1.58 ± 0.02	0.83 ± 0.09	0.25 ± 0.01	4.73 ± 0.07	0.77 ± 0.39	0.53 ± 0.04	26.81 ± 0.64	21.94 ± 0.46	4465 ± 246	<LOD	0.02 ± 0.03
DS35	3.35 ± 0.09	0.55 ± 0.04	0.111 ± 0.005	47.44 ± 0.88	1.54 ± 0.07	7.301 ± 0.004	7.55 ± 0.15	4.15 ± 0.07	4.53 ± 0.29	<LOD	0.31 ± 0.39

LOD for Ca = 0.14 µg/g; LOD for Cr = 0.17 µg/g; LOD for Cu = 0.04 µg/g; LOD for Fe = 1.55 µg/g; LOD for Zn = 0.26 µg/g; LOD for Pb = 0.08 µg/g; LOD for Cd = 0.01 µg/g.

**Table 5:** Results of the Dunn’s test conducted for the analysed data matrix (*p* < 0.05; *p* < 0.01; *p* < 0.001).

	GTE with Cr	GTE	GTE with Fruits Extracts	GTE with Cr and Fruit Extracts	Original Green Tea ^1^
GTE with Cr		Cr ^a^			K ^a^, Na ^a^, Cu ^b^, Fe ^b^, Mn ^c^, Zn ^b^
GTE	Cr ^a^			Cr ^a^	K ^c^, Mg ^c^, Na ^c^, Cr ^a^, Cu ^c^, Fe ^c^, Mn ^c^, Zn ^c^
GTE with fruits extracts					K ^a^, Fe ^b^, Mn ^b^
GTE with Cr and fruit extracts		Cr ^a^			Na ^b^, Zn ^a^
Original green tea	K ^a^, Na ^a^, Cu ^b^, Fe ^b^, Mn ^c^, Zn ^b^	K ^c^, Mg ^c^, Na ^c^, Cr ^a^, Cu ^c^, Fe ^c^, Mn ^c^, Zn ^c^	K ^a^, Fe ^b^, Mn ^b^	Na^b^, Zn ^a^	

^a^ *p* < 0.05; ^b^ *p* < 0.01; ^c^ *p* < 0.001. ^1^ the green tea data is based on [39] (GTE—dietary supplements containing green tea extracts; GTE with Cr-dietary supplements containing green tea extracts with chromium; GTE with Cr and fruit extracts-dietary supplements containing green tea extracts with chromium and fruit extracts; GTE with fruit extracts-dietary supplements containing green tea extracts with fruit extracts).

**Table 6:** Evaluation of RDA/AI [44] for the selected elements in the analysed dietary supplements.

Element	RDA ^a^/AI ^b^ Standard in the 19–51 Age Group [mg] ^1^		[%] RDA/AI Min-Max (According to Manufacturer’s Dosage Recommendations) ^2^	
	Capsule			
	Female	Man	Female	Man
Ca	1000 ^a^	1000 ^a^	<LOD-7	<LOD-7
K	3500 ^b^	3500 ^b^	0.002–0.681	0.002–0.681
Mg	320 ^a^	420 ^a^	0.003–1.612	0.002–1.231
Na	1500 ^b^	1500 ^b^	0.007–0.450	0.007–0.450
Mn	1.8 ^b^	2.3 ^b^	0.02–20.41	0.02–16.01
Fe	18 ^a^	10 ^a^	<LOD-156	<LOD-280
Cu	0.9 ^a^	0.9 ^a^	<LOD-8	<LOD-8
Zn	8 ^a^	11 ^a^	<LOD-235	<LOD-171
	**Tablet**			
	**Female**	**Man**	**Female**	**Man**
Ca	1000 ^a^	1000 ^a^	0.02–15.10	0.02–15.10
K	3500 ^b^	3500 ^b^	0.001–0.301	0.001–0.301
Mg	320 ^a^	420 ^a^	0.17–1.97	0.13–1.50
Na	1500 ^b^	1500 ^b^	0.01–1.13	0.01–1.13
Mn	1.8 ^b^	2.3 ^b^	0.11–29.50	0.09–23.10
Fe	18 ^a^	10 ^a^	<LOD-0.12	<LOD-0.22
Cu	0.9 ^a^	0.9 ^a^	0.01–0.95	0.01–0.95
Zn	8 ^a^	11 ^a^	<LOD-0.24	<LOD-0.17

^1^ based on [44]; ^2^ according to Table A1; ^a^ RDA—Recommended dietary allowances; ^b^ AI—Adequate intake

**Table 7:** Estimation of the tolerable monthly intake for Cd [%PTMI].

Code	Average Content of Cd [µg/Capsule/Tablet]	Average Intake of Cd [µg/Capsule/Tablet/30 Days]	Average Intake of Cd [µg/Monthly Dose] ^1^	%PTMI
DS8	0.020	0.60	1.20	0.03
DS9	0.008	0.24	0.48	0.01
DS11	0.008	0.24	0.96	0.01
DS14	0.037	1.11	2.22	0.06
DS21	0.046	1.38	1.38	0.08
DS22	0.010	0.30	0.90	0.02
DS30	0.015	0.45	0.90	0.03
DS31	0.032	0.96	1.92	0.05
DS33	0.078	2.34	4.68	0.13
DS34	0.025	0.75	3.00	0.04
DS35	0.105	3.15	9.45	0.18

^1^ Value calculated based on the manufacturer’s recommended dosage over a period of 30 days.

**Table 8:** Evaluation of the manufacturer’s declaration for Cr content.

Code	Declared Content of Cr [mg]	Average of Cr [µg/Capsule/Tablet]	Percentage of Declaration [%]
DS1	37.5	13.9	37.1
DS8	40.0	60.3	151
DS9	3.0	4.9	164
DS10	33.3	19.6	58.9
DS28	5.0	<LOD	ND
DS30	45.0	28.0	62.3
DS32	20.0	15.8	79.1
DS34	5.0	0.81	16.1

ND—no data; value cannot be calculated; LOD for Cr = 0.17 µg/g.

## Data Availability

All data is contained within the article.

## Appendix A

**Table A1:** Characteristics of the dietary supplements analysed based on information contained on the packaging.

Code	Composition	Pharmaceutical Form	Recommended Daily Dosage [Number of Capsules/Tablets]	Country of Origin
DS1	green tea extract (55% EGCG); guarana extract (50% caffeine); chromium; synephrine	Capsule	4	Poland
DS2	green tea extract (55% EGCG); caffeine	Capsule	2	Poland
DS3	green tea extract (EGCG); chili pepper; apple fibre (80%)	Capsule	1	-
DS4	*Camellia sinensis* green tea extract (EGCG)	Capsule	2	-
DS5	conjugated linoleic acid (CLA); green tea extract (EGCG); L-carnitine	Capsule	4	Poland
DS6	green tea extract (EGCG); caffeine; bitter orange fruit extract (synephrine); guarana seed extract, black pepper fruit extract	Capsule	2	Poland
DS7	conjugated linoleic acid (CLA); green tea extract (EGCG)	Capsule	4	Denmark
DS8	green tea extract (EGCG); caffeine; chromium; extract of Indian nettle root, black pepper fruit and cayenne pepper	Capsule	2	-
DS9	green tea extract (EGCG); caffeine; green coffee extract; chromium; iodine	Capsule	2	USA
DS10	green tea leaf extract; L-tyrosine; L-carnitine; caffeine; ginger root extract; African mango seed extract; ginseng root; black peppercorn extract; chromium	Capsule	3	Poland
DS11	*Camellia sinensis* green tea extract	Capsule	4	-
DS12	green tea leaf extract	Capsule	1	Poland
DS13	green tea leaf extract (EGCG); L-carnitine	Capsule	4	Poland
DS14	green tea; cider vinegar extract	Capsule	2	Poland
DS15	green tea leaf extract (EGCG)	Capsule	1	USA
DS16	green tea leaf extract (EGCG)	Capsule	3	Germany
DS17	green tea leaf extract; CLA; L-carnitine	Capsule	6	UK
DS18	green tea (*Camellia sinensis*) leaf extract (EGCG)	Capsule	1	USA
DS19	green tea leaf extract; vitamin C	Capsule	1	USA
DS20	green tea leaf extract (EGCG)	Capsule	1	USA
DS21	leafy green tea	Capsule	1	USA
DS22	green tea leaf extract (EGCG)	Capsule	3	UK
DS23	green tea leaf extract (EGCG)	Capsule	1	USA
DS24	decaffeinated green tea extract (EGCG)	Capsule	1	USA
DS25	conjugated linoleic acid (CLA); L-carnitine; *Camellia sinensis* green tea extract	Capsule	1	Poland
DS26	green tea extract	Capsule	2	USA
DS27	nettle herb extract; green tea leaf extract; prickly pear extract; grape seeds; dandelion root; goldenrod herb	Tablet	1	Poland
DS28	green tea extract; chromium; L-carnitine	Tablet	2	Czech Republic
DS29	green tea leaf extract	Tablet	1	Poland
DS30	green tea extract; L-carnitine; chromium; chlorella; nettle leaf extract	Tablet	2	Poland
DS31	green tea extract (EGCG); caffeine; extract of nettle; cayenne pepper; green coffee	Tablet	2	Poland
DS32	green tea leaf extract; bitter orange extract (synephrine); *Paraguayan ginkgo* leaf extract; long fiddle leaf extract; chromium	Tablet	2	Poland
DS33	green tea extract (*Camellia sinensis*); African mango extract; caffeine	Tablet	2	UK
DS34	L-carnitine; green tea leaf extract; CLA; *Garcinia cambogia* fruit extract	Tablet	4	-
DS35	green tea extract (*Camellia sinensis*); acai extract	Tablet	3	UK

EGCG—epigallocatechin gallate; CLA—concentrated linoleic acid.

**Table A2:** Validation parameters for the FAAS method.

Element	Accuracy [%]	Precision [%]	LOD [µg/g]	LOQ [µg/g]
Na	98	10.21	0.06	0.19
K	108	2.06	0.04	0.11
Ca	92	4.17	0.14	0.43
Mg	99	0.59	0.01	0.03
Mn	112	1.67	0.01	0.04
Fe	107	0.63	1.55	4.64
Zn	107	4.31	0.26	0.78
Cr	89	0.02	0.17	0.51
Cu	99	1.42	0.04	0.11
Cd	105	3.48	0.01	0.03
Pb	95	5.61	0.08	0.25
